# The impact of endoscopic thoracic sympathectomy on sudomotor function in patients with palmar hyperhidrosis

**DOI:** 10.1007/s10286-020-00685-2

**Published:** 2020-04-27

**Authors:** Naomi Hirakawa, Ikuyo Higashimoto, Ayako Takamori, Eri Tsukamoto, Yuhei Uemura

**Affiliations:** 1 Department of Pain Clinic & Palliative Care Medicine, Department of the Anesthesiology & Critical Care Medicine, Saga University Hospital, 5-1-1, Nabeshima, Saga, 849-8501 Japan; 2 Department of Anesthesiology, JCHO Saga Central Hospital, Saga, Japan; 3 Clinical Research Center, Saga University Hospital, Saga, Japan; 4 Department of Pain Clinic & Palliative Care Medicine, Saga University Hospital, 5-1-1, Nabeshima, Saga, 849-8501 Japan; 5 Uemura Pain Clinic, Fukuoka, Japan

**Keywords:** Endoscopic thoracic sympathectomy, Palmar hyperhidrosis, Peak sweat rate, Perspiration meter, Sweat volume

## Abstract

**Purpose:**

When performing endoscopic thoracic sympathectomy (ETS) in palmar hyperhidrosis patients, a device can be used to measure sweat volume pre- and postoperatively in order to assess indications and treatment effects. In this study, we measured changes in the dynamics of sweating in hyperhidrosis patients pre- and postoperatively and compared the values with those in healthy subjects without hyperhidrosis.

**Methods:**

The patient group comprised 25 persons with palmar hyperhidrosis who were scheduled for ETS. The dynamics of sweating was measured at 1 day prior to surgery and at 2 days postoperatively, in 18 patients at > 1 year postoperatively in another palmar hyperhidrosis group, and in 20 healthy subjects without hyperhidrosis. A device for measuring local sweat volume was applied at the thenar eminence of both palms. Indicators established were basal sweat rate (BSR; mg/min/cm^2^), peak sweat rate (PSR; mg/min/cm^2^) during mental stress (sympathetic sweating response), sweat volume (SV), and sweat time (ST; s).

**Results:**

After surgery, all of the indicators were significantly reduced in hyperhidrosis patients and there was very little response to mental stress. The subgroup of these patients assessed at > 1 year after ETS showed a trend of increased BSR similar to that of healthy subjects. These changes did not correlate with the extent of the removal surgery. Preoperatively, hyperhidrosis patients had significantly greater BSR, PSR, and SV and longer ST than healthy subjects.

**Conclusion:**

All of the sweating parameters were increased in palmar hyperhidrosis patients prior to surgery. Immediately after ETS, all these parameters were significantly reduced. At > 1 year after ETS, the BSR had increased to a level similar to that of the healthy volunteers, although PSR did not respond to mental stress.

## Introduction

Sweating can be generally classified into thermal and emotional sweating. Thermal sweating, which involves almost all of the skin of the body, is the body’s response to heat exposure, while emotional sweating, which involves primarily the palms of the hands and soles of the feet, is caused by mental tension or emotional fluctuations and is independent of ambient temperature. In response to mental arousal, the sweating of the palms increases immediately. The lateral horn of the spinal cord plays a key role in palmar sweating, sending signals to the palmar sweat glands through cholinergic sympathetic postganglionic fibers to control sweat volume (SV) [1]. The primary mechanisms of emotional sweating involve the prefrontal area of the cerebral cortex, the limbic system, and the hypothalamus. Emotional sweating of the palms is controlled by signals sent to the eccrine glands of the palms through the cholinergic sympathetic nerves in the lateral horn of the spinal cord from the eighth cervical vertebra to the sixth thoracic vertebra (T6) [1–3].

Palmar hyperhidrosis is a condition in which a person experiences excessive palmar sweating, without any underlying disease. Palmar hyperhidrosis affects 1–3% of the population, and its cause remains unknown [4]. Patients suffering from palmar hyperhidrosis not only experience a decreased quality of life due to mental distress, but also show reduced labor productivity.

Severe forms of palmar hyperhidrosis are resistant to local treatment and drug therapy. In such cases, endoscopic thoracic sympathectomy (ETS), a surgical procedure used to treat excessive sweating in certain parts of the body, can be performed if strongly desired by the patient, after a thorough explanation of the possible complications and sequelae, such as compensatory hyperhidrosis [5]. During ETS, a perspiration meter is used to measure SV before and following the surgical procedure in order to assess indications and treatment effects. Compensatory sweating is a potential secondary effect of ETS which reduces patient satisfaction, and this condition has been the focus of many studies [6–10]. However, these studies have typically only looked at changes in palmar sweating before and immediately after the surgery. We hypothesized that sweating dynamics would change over a long period after surgery compared to the immediate post-surgery period.

The aim of this study was to evaluate quantitative and comparative assessments of the dynamics of sweating in patients with palmar hyperhidrosis before and after ETS.

## Subjects and methods

This study was conducted according to the guidelines laid down by the Helsinki Declaration of 1964 and its later amendments, and all procedures involving human subjects were approved by the Ethical Review Board for Clinical Research of Saga University Hospital. Consent was obtained from all participants.

Patients with palmar hyperhidrosis who underwent ETS at the second, third, or second and third thoracic vertebrae (T2, T3, and T2 + T3, respectively) at our department between 2006 and April 2017 and healthy subjects without hyperhidrosis were included in this study. Patients with comorbidities, healthy subjects with hyperhidrosis or comorbidities, and patients who had relapsed after surgery were excluded. Diagnosis of palmar hyperhidrosis was made according to Hornberger’s diagnostic criteria [11]. The sample size was established based on the number of patients for whom we performed sweat measurements during a period of > 1 year after the surgery. None of the patients with palmar hyperhidrosis included in the study had any comorbidity. The participants in the study were classified into the following groups:

- Group PH: Palmar hyperhidrosis patients (25 patients, 50 sides [right and left, respectively]) who had undergone ETS in the past year and whose SV was measured 1 day before surgery. Patients in group PH were the same as those in group A.
- Group A: Palmar hyperhidrosis patients who had undergone ETS in the past year and whose SV was measured 1 day before surgery (Group PH) and 2 days after surgery (25 patients, 50 sides).
- Group B: Palmar hyperhidrosis patients who had undergone ETS for > 1 year earlier (postoperative period: 43.6 ± 17.4 months) (18 cases, 36 sides). (These patients were different from those in group A and showed successful effects immediately after ETS.) Contact had been maintained with these patients, and they were willing to undergo perspiration measurements at > 1 year after ETS.
- Group C: Healthy subjects without palmar hyperhidrosis (20 cases, 40 sides).

SV was continuously measured at the thenar eminence of the bilateral palms using a flow-control perspiration meter with a ventilation capsule (SKN 2000^Ⓡ^; SKINOS Co. Ltd. Nagoya, Japan). All measurements were taken at the outpatient pain clinic (room temperature 24–26 °C, humidity 55–65%) with the participants in a sitting position. Once the participant had rested for a minimum of 5 min and SV had stabilized on the left and right sides, he/she was subjected to a mental stress, in the form of a mental arithmetic task (serial subtraction by seven, beginning from 1000; for example, 1000 − 7, 993 − 7, 986 − 7) for 2 min. Following this 2-min-long mental stress, the SV was measured again on both sides until the pre-mental stress level had been achieved. The following parameters, as measured by the perspiration meter, were also recorded for quantitative analysis of sweating: basal sweat rate (BSR; mg/min/cm^2^), peak sweat rate (PSR; mg/min/cm^2^), sweat rate (SR) at probe attachment (SR start; mg/min/cm^2^), SV (mg/cm^2^), and sweat time (ST; s).

### Statistical analysis

Data are shown as the mean ± standard deviation. The unpaired Student’s *t* test was used to compare parameters between group PH and group C, and the paired Student’s *t* test was used to compare parameters between group PH and group A. Additionally, multiple analysis was performed among groups A, B, and C using Dunnett’s test. The JMP Pro 14 software program (SAS Institute, Cary, NC, USA) was used for the statistical analysis. The significance level was set at *P* < 0.05.

## Results

No significant differences were found in terms of age or sex in each group (Table 1). All sweating parameters of group PH were significantly higher than those of group C (*P* < 0.0001). (Table 2). All the parameters of group PH were also significantly higher than those of group A (*P* < 0.0001). Table 3).

**Table 1:** Background of study participants

Study group characteristics^a^	Study group^b^		
	A	B	C
Male-to-female ratio (M:F)	10:5	4:16	6:14
Age (years)^c^	24.6 ± 10.5 [15–65]	28.7 ± 14.8 [14–67]	33.6 ± 9.3 [17–50]

^a^There were no significant intergroup differences

^b^Group A: Palmar hyperhidrosis patients who had undergone endoscopic thoracic sympathectomy (ETS) in the past year and whose sweat volume (SV) had been measured 1 day before surgery (group PH) and 2 days after surgery. Group B: Palmar hyperhidrosis patients who had undergone ETS > 1 year earlier, with successful effects immediately after ETS, and for whom perspiration measurements had been made at > 1 year after ETS. Group C: Healthy subjects without palmar hyperhidrosis. For more details, see list in section “Subjects and methods”

^c^Age is given as the mean ± standard deviation (SD) with the range in square brackets

**Table 2:** Comparison of the sweat parameters between group PH and group C

Sweat parameters	Study group^a^	
	Group PH	Group C
BSR (mg/min/cm^2^)	1.03 ± 0.42	0.21 ± 0.08
PSR (mg/min/cm^2^)	2.02 ± 0.43	0.70 ± 0.45
SV (mg/cm^2^)	5.00 ± 2.57	0.23 ± 0.13
ST (s)	218.44 ± 91.71	34.40 ± 13.22
SR start (mg/min/cm^2^)	1.97 ± 0.71	0.76 ± 0.33

Values are presented as the mean ± SD

*BSR* Basal sweat rate,* PSR* peak sweat rate,* SR start* sweat rate (SR) at probe attachment,* ST* sweat time

Significant differences were found for all sweating parameters between group PH and group C (*P* < 0.0001)

^a^Group PH: Palmar hyperhidrosis patients who had undergone ETS in the past year and whose SV was measured 1 day before surgery. Groups PH and A comprised the same patients. For more details, see list in section Subjects and methods

**Table 3:** Comparison of the sweat parameters between group PH and group A

Sweat parameters	Study group	
	Group PH	Group A
BSR (mg/min/cm^2^)	1.03 ± 0.42	0.06 ± 0.05
PSR (mg/min/cm^2^)	2.02 ± 0.43	0.14 ± 0.14
SV (mg/cm^2^)	5.00 ± 2.57	0.03 ± 0.04
ST (s)	218.44 ± 91.71	11.64 ± 10.3
SR start (mg/min/cm^2^)	1.97 ± 0.71	0.12 ± 0.08

Values are presented as the mean ± SD

Significant differences in all the parameters were also found between group PH and Group A (*p* < 0.0001)

**Fig. 1:**
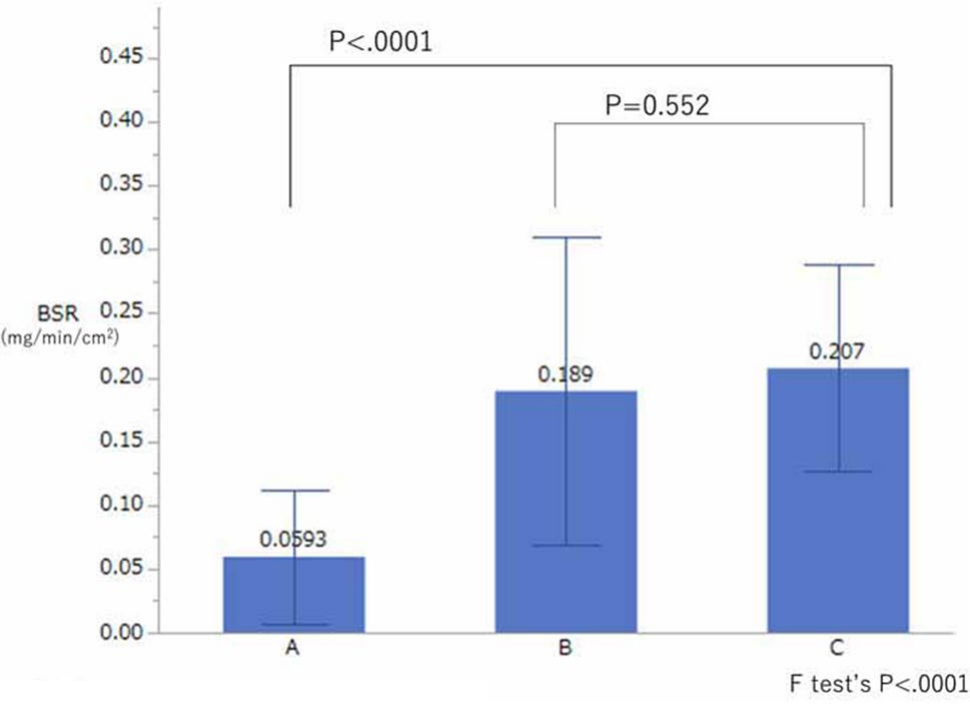
Comparison of the basal sweat rate (*BSR*) in groups A, B, and C. The BSR in group A was significantly different from that in group C (*P* < 0.001, estimated by Dunnett’s test), but there was no significant difference in BSR between group B and group C. Group A: Palmar hyperhidrosis patients who had undergone endoscopic thoracic sympathectomy (ETS) in the past year and whose SV was measured 1 day before the surgery (group PTH) and 2 days after the surgery (25 patients, 50 sides). Group B: Palmar hyperhidrosis patients who had undergone ETS > 1 year earlier, with successful effects immediately after ETS, and for whom perspiration measurements had been made at > 1 years after ETS. (Group A and group B were different patients). Group C: Healthy subjects without palmar hyperhidrosis (20 patients, 40 sides)

**Fig. 2:**
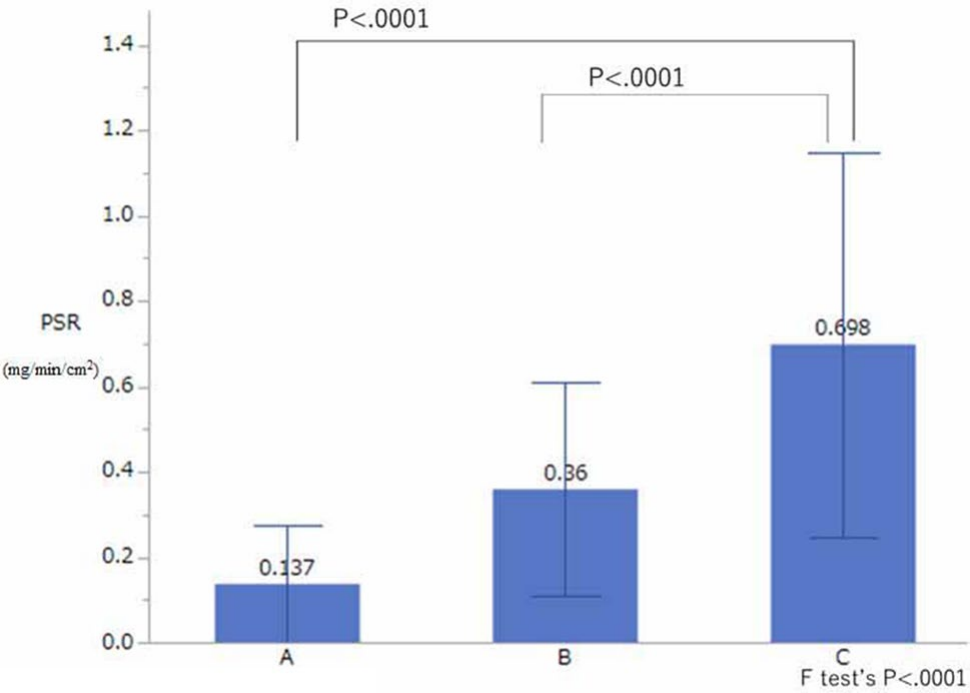
Comparison of the peak sweat rate (*PSR*) in groups A, B, and C. The PSR in group A and group B was significantly different from that in group C (both *P* < 0.001, estimated by Dunnett’s test). See caption to Fig. 1 for descriptions of groups

The BSR in group A was significantly lower than that in group C (*P* < 0.001), but there was no significant difference in the BSR between group B and group C (Fig. 1). The PSR of group A and group B was significantly lower than that of group C (both *P* < 0.001) (Fig. 2).

**Fig. 3:**
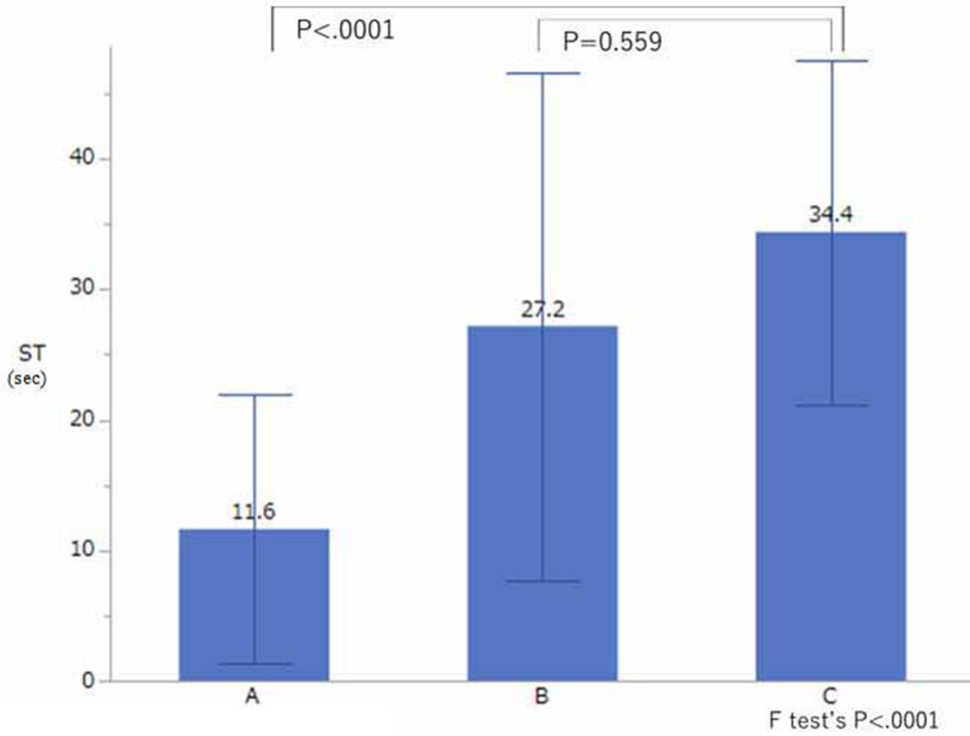
Comparison of the sweat time (ST) in groups A, B, and C. The ST during the mental arithmetic task was significantly different between group A and group C (*P* < 0.001, estimated by Dunnett’s test), but no significant difference was found between group B and group C

The ST during the mental arithmetic task in Group A was significantly shorter than that during the mental arithmetic task in Group C; however, no significant difference in ST during mental arithmetic task was found between group B and group C (Fig. 3).

**Fig. 4:**
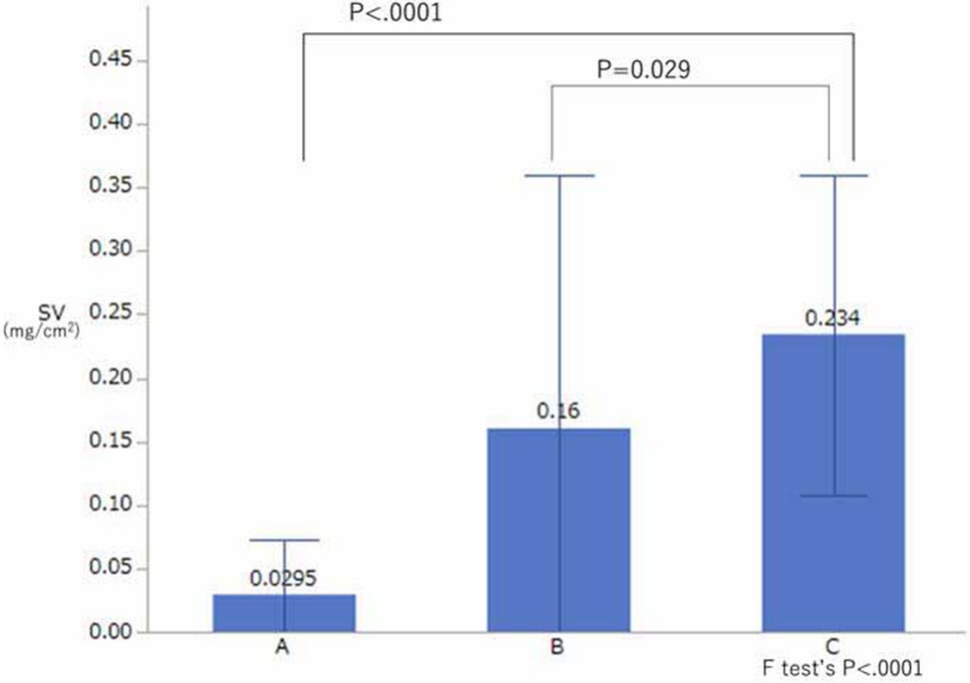
Comparison of the sweat volume (*SV*). The SV in group A was significantly different from that in group C (*P* < 0.001, estimated by Dunnett’s test), but the SV of group B and group C was not significantly different

The SV in group A was significantly less than that in group C (*P* < 0.001), but it was not significantly different between group B and group C (Fig. 4).

## Discussion

In the study reported here we compared sweating parameters in healthy volunteers with those of patients with palmar hyperhidrosis at 1 day prior to ETS, 2 days after ETS, and at > 1 year after ETS. Before surgery, patients had greater BSR, PSR, and SV and longer ST than the healthy controls. We found that SV was significantly reduced in hyperhidrosis patients postoperatively and was not markedly triggered by mental stress at this time. At > 1 year after ETS, patients tended to show increased BSR.

Hyperhidrosis refers to a condition in which the afficted person sweats more than normal. However, as even healthy individuals differ in terms of sweating, diagnosing local hyperhidrosis requires an objective evaluation and quantitative assessment of sweating utilizing a highly reliable method. Quantitative testing of the sweating function is also useful for evaluating the temporal aspects of a sweating disorder. The SKN-2000 device used in the present study is a flow volume-compensating ventilated capsule perspiration meter which works by bringing room air into the capsule and then measuring the difference in humidity between before and after the air is brought into the capsule. It contains a servo system that controls the volume of airflow, depending on the SV, which allows high responsiveness and objective measurement of hyperhidrosis [12].

To measure emotional sweating, we used a mental arithmetic problem to induce stress, as has been reported in an earlier study [13]. A previous study showed that all stimuli presented were able to induce increased sweating in patients with palmar hyperhidrosis compared to non-hyperhidrosis individuals and that the difference caused by mental arithmetic, as an emotional stimulus, was the largest [14]. Presenting a similar load repeatedly during a single testing session can cause a “habituation phenomenon,” in which responses become increasingly unclear; therefore, the duration of the mental arithmetic task was set at 2 min in our study. Okada et al. [15] reported on the types of emotional sweating when emotional stress was induced in 124 healthy individuals. These authors classified sweating phenomena in these healthy individuals with a focus on the baseline stability at rest and responses to basic loading stimuli and noted a workload selectivity and an increase in SV and in the rate of sweat suppression during the response to stress. They found that the proportion of individuals with a stable baseline and of those with an unstable baseline at rest were nearly equal. Moreover, most individuals tended to respond more to mental stress than to exercise load, and in most individuals the SV was large and the recovery time to the baseline was fast [14]. In the present study, baseline sweating of most of the healthy volunteers was stable and the recovery time to baseline was also fast.

Among previous studies that measured sweating before and immediately after ETS, Ishy et al. observed changes in sweating over time. These authors used a VapoMeter to compare transepidermal water loss up to 12 months after resection of either the T3 or the T4 sympathetic ganglia and found that palmar sweating was significantly reduced in both groups 12 months after the surgery [7]. They also compared the SV between preoperative palmar hyperhidrosis patients and patients without hyperhidrosis and confirmed that SV was fourfold higher in patients with palmar hyperhidrosis. Nevertheless, to date no studies have reported assessment of the individual components of sweat measurement or changes pre- and postoperatively in hyperhidrosis patients. To our knowledge, our study is the first to quantitatively assess each parameter of the pre-and postoperative states of palmar hyperhidrosis patients.

It is generally considered that a prolonged ST is more characteristic of preoperative hyperhidrosis patients than a high PSR. However, in our study we observed elevated BSR, prolonged ST, and increased PSR in palmar hyperhidrosis patients challenged with the mental stress task peroperatively (1 day prior to ETS). Most of the palmar hyperhidrosis patients presented an excessive sweating of the palms (1.97 ± 0.72 mg/min/cm^2^) when the measurement was made preoperatively, whereas immediately after the surgery (2 days) as well as for some time thereafter the sweating was significantly lower than that of healthy individuals at the beginning of the examination. Okada et al. observed a sharp peak in sweating in palmar hyperhidrosis patients in response to various types of stress, within a short period. These response patterns did not differ for physical or emotional stimuli, and they were considered to represent a “pan-reactive type” of response [15]. Since we did not provide physical stimulations in our study, we analyzed responses to an emotional stimulus only. We found that although subjects without hyperhidrosis also responded to mental stress, they recovered quickly and no elevated SV was observed; in contrast, while palmar hyperhidrosis patients also responded to mental stress, they showed prolonged ST and increased SV.

We observed that immediately after ETS, both the BSR and PSR decreased markedly in patients, and no changes in response to mental stress were observed. When these parameters were measured > 1 year after ETS, the BSR of the palmar hyperhidrosis patients was not significantly different from that of healthy individuals, and all subjects continued to be nonresponsive to mental stress. To summarize, a small increase in the BSR, which is not related to sympathetic nervous system stimulation, was observed in palmar hyperhidrosis patients immediately after surgery, whereas at a much later date after surgery (> 1 year), the BSR had recovered to a level similar to that of healthy individuals. However, these patients showed less response to emotional stress than healthy individuals.

This study has a number of limitations. First, while it is possible to measure a very small change in SV simply, quickly, and non-invasively using a perspiration meter, a method that is useful for quantitative diagnosis when the aim is to determine the severity, distribution, and treatment effects in palmar hyperhidrosis, this approach has a number of drawbacks, including individual differences in the effects of mental stress and variation in measured values due to measurement site and individual differences. Second, only the evaluation of local sweating was possible; sweating across the whole body cannot be measured. Third, we set the duration of the mental arithmetic task at 2 min; this is shorter than the standard length of mental stress protocols, which is 5 min. Fourth, the sample size was small due to the inability to follow up patients in the long term; thus, a study with a long-term follow-up of patients should be performed. It should be noted that determining the extent to which palmar SR should be reduced is a challenge that needs to be addressed in future research.

All parameters of sweating were higher in palmar hyperhidrosis patients tested preoperatively, while immediately after ETS, all of the parameters significantly reduced. At > 1 year after ETS, the BSR had increased to a level similar to that of the healthy volunteers, although the PSR did not respond to mental stress.
